# Performance of Acute Physiology and Chronic Health Evaluation (APACHE II), Simplified Acute Physiology Score (SAPS III), and Sequential Organ Failure Assessment (SOFA) Scores in a Medical Intermediate Care Unit

**DOI:** 10.7759/cureus.89010

**Published:** 2025-07-29

**Authors:** Pedro N Ferreira, Joana A Duarte, João Fustiga, Marta Anastácio

**Affiliations:** 1 Internal Medicine Department, São Francisco Xavier Hospital, Lisbon, PRT; 2 Physiopathology, Faculdade de Medicina Universidade de Lisboa, Lisbon, PRT; 3 Intermediate Medical Care Unit, Internal Medicine Department, São Francisco de Xavier Hospital, Lisbon, PRT; 4 Intensive Care Department, São Francisco Xavier Hospital, Lisbon, PRT

**Keywords:** apache ii, imcu, intermediate medical care unit, mortality prediction, outcome prediction, saps iii, severity scores, sofa

## Abstract

Methods: A retrospective observational study was conducted at the intermediate medical care unit (IMCU) in São Francisco Xavier Hospital, a Portuguese hospital, in the period from January to December 2019. Patients with incomplete records or IMCU stays shorter than 24 hours were excluded. Scores were calculated using MDCalc, and statistical analysis was performed using SPSS version 27.

Results: Of the 211 patients included in this analysis, 14.69% (n=31) died, with 16.1% (n=5) of these deaths occurring in the IMCU. The average age of non-survivors was significantly higher at 76.26 years (p < 0.001). Acute Physiology and Chronic Health Evaluation (APACHE II) and Simplified Acute Physiology Score (SAPS III) scores demonstrated good discrimination, with AUCs of 0.79 and 0.83, respectively, but both scores tended to overestimate mortality. Sequential Organ Failure Assessment (SOFA) showed the lowest discrimination performance with an Area Under the Curve (AUC) of 0.74.

Conclusion: APACHE II and SAPS III provided good mortality prediction in the IMCU, with SAPS III showing the highest AUC. However, both scores overestimated mortality, indicating the need for calibration for IMCUs. The SOFA score was less effective due to its focus on organ failure. Further research is needed to adapt these models or develop new ones tailored specifically for IMCUs to improve predictive accuracy and clinical utility.

## Introduction

Intermediate medical care units (IMCUs) are unique structures that fill the gap between general wards and intensive care units. They are specially designed to ensure the surveillance of complex or unstable patients whose monitoring, therapeutic, and diagnostic needs exceed the capabilities of general wards but do not require admission to an intensive care unit (ICU) [1,2]. The primary goal of escalating care is to prevent complications, anticipate clinical deterioration, and reduce mortality rates. In the ICU, several validated scores predict patient outcomes; however, due to the heterogeneity of IMCUs and their patients across institutions, no single score has been categorically validated to predict outcomes in IMCUs [1-12]. Consequently, ICU-validated scores are often adapted for IMCU use without exactly knowing how accurate they are in this setting [1,2,4,12].

The Acute Physiology and Chronic Health Evaluation (APACHE) II score is widely used for outcome prediction and encompasses 12 physiological variables weighted according to their relative impact. APACHE II provides an estimated mortality rate based on the total score and shows good discrimination when applied to IMCUs [1,12].

The Simplified Acute Physiology Score (SAPS) III, which includes 20 variables divided into three major categories (pre-admission patient information, admission circumstances, and physiological variables), was developed from global data and demonstrated good discrimination and calibration in ICU patients [11]. However, SAPS III tends to overestimate mortality in IMCUs [1,2,4,12].

The Sequential Organ Failure Assessment (SOFA) score was initially designed to quantify organ dysfunction in six physiological systems (neurological, respiratory, cardiovascular, hematological, hepatic, and renal) rather than predict mortality [5,10]. Nevertheless, due to the association between cumulative organ failure and mortality, SOFA is widely used for mortality prediction.

## Materials and methods

Objective

This study aims to compare the APACHE II, SAPS III, and SOFA severity scores to determine which score has the highest predictive value for mortality when applied in an IMCU.

Background

The staff of the IMCU at São Francisco Xavier Hospital, in Portugal, decided to start calculating severity scores (APACHE II, SAPS III, and SOFA) in 2018, in order to try to estimate mortality rates and evaluate the quality of care provided to their patients. This retrospective observational study, conducted between January and December of 2019, aims to determine which score has the highest predictive value for mortality in an IMCU. The analysis refers to the last pre-COVID-19 pandemic year for which data was finalized. Patients with incomplete records or those admitted to the IMCU for less than 24 hours were excluded. Formal consent was not required as data collection was performed through clinical records, and the Hospital Ethics Council's opinion was deemed unnecessary.

Data collection

All patients' clinical data were collected retrospectively from an institutional database (SClinico). The following variables were analyzed: age, sex, previous degree of autonomy (using the Barthel Index), location prior to admission, destination after discharge, length of stay in the IMCU, total length of hospital stay, IMCU mortality, and overall hospital mortality. During hospitalization, the presence of sepsis - defined as organ dysfunction caused by a dysregulated host response to infection per Sepsis-3 guidelines - was also analyzed. The SOFA score was calculated at admission for all patients, while APACHE II and SAPS III were calculated 24 hours after admission using the MDCalc online calculator (Healthcare Technology; New York, NY, USA). Patient’s records were reviewed 30 days after hospital discharge.

Statistical analysis

Statistical analysis was performed using SPSS version 27 for Windows (SPSS Inc., Chicago, IL, USA). Continuous variables were reported as mean and standard deviation, while categorical variables were reported as total number and percentages. Continuous variables were first evaluated using the Shapiro-Wilk normality test; with a p-value <0.05, nonparametric tests were chosen. The Mann-Whitney U test established correlations between groups and continuous variables. Categorical variables were compared using Fisher’s exact test and the chi-square test. A p-value <0.05 was considered statistically significant. Discrimination was assessed using receiver-operating characteristic (ROC) curves. Area under the curve (AUC) values >0.75 were considered satisfactory, >0.8 good, and >0.9 very good.

## Results

In 2019, 212 patients were admitted to the IMCU, with one patient excluded for having a hospital stay of less than 24 hours. Of the remaining 211 patients, 46.9% (n=99) were male, and 53.1% (n=112) were female, with no significant gender difference (Pearson chi-square of 0.36) (Table 1).

**Table 1:** Characteristics of the Population Admitted to the IMCU APACHE II: Acute Physiology and Chronic Health Evaluation II; ER: emergency department; ICU: intensive care unit; IMCU: intermediate medical care unit; SAPS III: simplified acute physiology score III; SOFA: sequential organ failure assessment ^#^A p-value <0.05 was considered statistically significant. ^$^Fisher's exact test. ^€^Chi-square test. ^¥^Mann-Whitney U test. *data represented as total number (N) and percentage (%). ^+^data has been represented as mean and standard deviation (±SD).

	Surviving (N=180)	Non-surviving (N=31)	p-value^#^
Gender*			
Male	86 (47.8%)	13 (41.9%)	0.566^$^
Female	94 (52.2%)	18 (58.1%)	0.566^$^
Age^+^	65.27 (18.19)	76.26 (14.84)	0.001^¥^
Barthel index^+^	79.89 (30.63)	48.71 (41.31)	<0.001^¥^
Length of stay (days)^+^			
IMCU	5.18 (3.8)	6.84 (6.73)	0.492^¥^
Global	19.87 (30.65)	28.61 (31.76)	0.183^¥^
Location prior to admission*			
ER	78 (43.3%)	13 (41.9%)	0.066^€^
Medicine ward	22 (12.2%)	9 (29.0%)	
ICU	60 (33.3%)	6 (19.4%)	
Non-medical ward	11 (6.1%)	3 (9.7%)	
Other hospital	9 (5.0%)	0	
Destination*			
Death in IMCU	0	5 (16.1%)	0.066^€^
Home	20 (11.1%)	0	
Medicine ward	129 (71.1%)	20 (64.5%)	
ICU	8 (4.4%)	6 (19.4%)	
Non-medical ward	18 (10.0%)	0	
Other hospital	5 (2.8%)	0	
Sepsis*	51 (28.3%)	17 (54.8%)	0.006^$^
Primary diagnosis*			
Shock	59 (32.8%)	17 (54.8%)	0.068^€^
Cardiovascular	38 (21.1%)	1 (3.2%)	
Respiratory	35 (19.4%)	7 (22.6%)	
Endocrine	13 (7.2%)	0	
Digestive	9 (5.0%)	2 (6.5%)	
Genitourinary	6 (3.3%)	0	
Others	20 (11.1%)	4 (12.9%)	
Outcome prediction model			
SOFA			<0.001^¥^
Total score^+^	2.7 (2.3)	4.7 (2.3)	
APACHE II			
Total score^+^	10.9 (6.94)	17.7 (6.95)	
Predicted mortality (%)	15.9%	29.6%	
SAPS III			
Total score^+^	44.7 (3.47)	65.2 (19.1)	
Predicted mortality (%)	22%	49.1%	

The average age was 66.9 years (SD 18.2), with non-survivors recording a higher average age of 76.26 years, which was statistically significant (p 0.001) and a relevant predictor of mortality (Table 2). Similarly, greater dependence at admission, as indicated by higher Barthel Index scores, was more common among non-survivors (p<0.001).

**Table 2:** Mann-Whitney U Test Results APACHE II: Acute Physiology and Chronic Health Evaluation II; IMCU: intermediate medical care unit; LOS: length of stay; Mort: predicted mortality; SAPS III: simplified acute physiology score III; Sig: significance; SOFA: sequential organ failure assessment *The significance level is 0.050 ^#^Asymptotic significance is displayed

Null Hypothesis	Test	Sig.*^#^	Decision
1. The distribution of Age is the same across categories of non-surviving.	Independent-Samples Mann-Whitney U Test	0.001	Reject the null hypothesis.
2. The distribution of LOS_IMCU is the same across categories of non-surviving.	Independent-Samples Mann-Whitney U Test	0.492	Retain the null hypothesis.
3. The distribution of LOS_Global is the same across categories of non-surviving.	Independent-Samples Mann-Whitney U Test	0.183	Retain the null hypothesis.
4. The distribution of the Barthel Index is the same across categories of non-surviving.	Independent-Samples Mann-Whitney U Test	0.000	Reject the null hypothesis.
5. The distribution of SOFA is the same across categories of non-surviving.	Independent-Samples Mann-Whitney U Test	0.000	Reject the null hypothesis.
6. The distribution of APACHE_II is the same across categories of non-surviving.	Independent-Samples Mann-Whitney U Test	0.000	Reject the null hypothesis.
7. The distribution of Mort_APACHE II is the same across categories of non-surviving.	Independent-Samples Mann-Whitney U Test	0.000	Reject the null hypothesis.
8. The distribution of SAPS III is the same across categories of non-surviving.	Independent-Samples Mann-Whitney U Test	0.000	Reject the null hypothesis.
9. The distribution of Mort_SAPS III is the same across categories of non-surviving.	Independent-Samples Mann-Whitney U Test	0.000	Reject the null hypothesis.

Length of stay, whether in the IMCU or overall, showed no significant relationship with mortality. The presence of sepsis was more common among non-survivors and was statistically significant (p = 0.006). Shock was more frequent among non-survivors but was not statistically significant, even though this subgroup showed a higher average age (78.1 years) and higher dependence according to the Barthel Index (47.1).

Only one cardiovascular diagnosis was registered in the non-survivor subgroup, which corresponded to a case of pulmonary embolism, compared to twenty-seven cases in the survivor subgroup. All deaths related to the respiratory system were due to pneumonia, with an average age of 87 years and a Barthel Index of 27.

Regarding the main groups of diagnosis of the admitted patients in the IMCU, the most common ones were shock, cardiovascular, and respiratory. The most common type of shock was septic shock (N=67), followed by anaphylactic (N=4), hypovolemic (N=3), and cardiogenic shock (N=2).

Within the cardiovascular group, the most frequent diagnosis was pulmonary embolism (n=28) followed by heart failure (N=6). Concerning the respiratory group of admission, the most prevalent diagnosis was bacterial pneumoniae with respiratory failure (n=27) and chronic obstructive lung disease exacerbation (n=11). The recorded mortality rate was 14.69% (N=31), with 16.1% (N=5) of deaths occurring in the IMCU.

As shown in Table 2, the scores under study positively correlated with mortality. Among the general population, the predicted mortality by APACHE II score (18%) was the closest to the mortality observed in our population (Table 3).

**Table 3:** Scores of General Population (n=211) APACHE II: acute physiology and chronic health evaluation II; SAPS III: simplified acute physiology score III; SOFA: sequential organ failure assessment *data represented as mean and standard deviation (±SD)

Outcome Prediction Model	General Population Scores
SOFA	
Total score*	3 (2.3)
APACHE II	
Total score*	11.9 (6.9)
Predicted mortality (%)	18%
SAPS III	
Total score*	47.6 (19.1)
Predicted mortality (%)	25.8%

**Figure 1:**
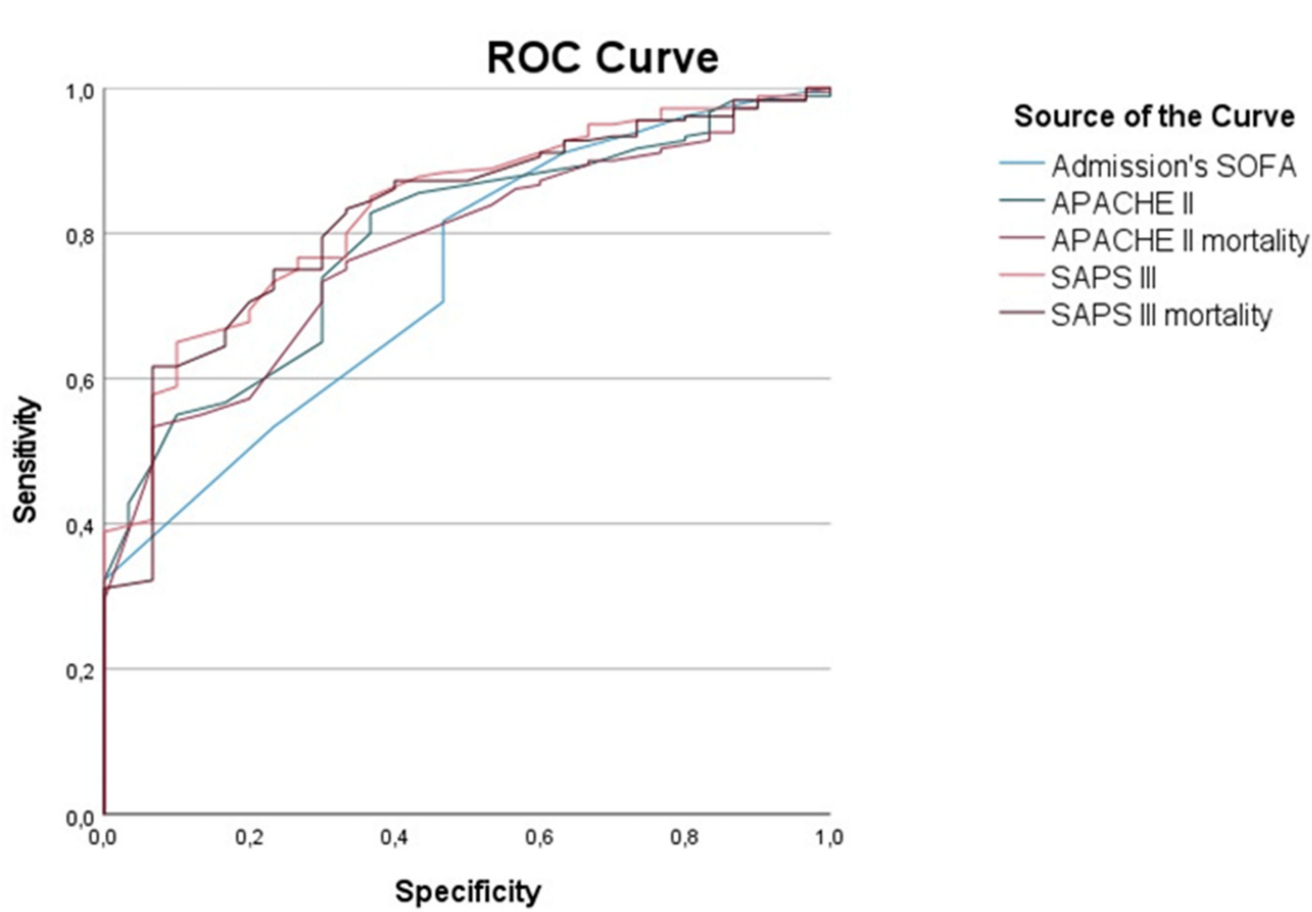
Receiver-operating characteristic curve for SOFA, APACHE II, and SAPS III SOFA: Sequential Organ Failure Assessment; APACHE II: Acute Physiology and Chronic Health Evaluation; SAPS III: Simplified Acute Physiology Score

ROC curve analysis (Table 4 and Figure 1) showed that both APACHE II and SAPS III demonstrated good discrimination value, with SAPS III having the highest predictive value for mortality. Nevertheless, SAPS III significantly overestimated mortality, with the predicted mortality for the general population being 25.8% and for the non-survivors being 49.1%.

**Table 4:** Area Under the Curve (AUC) APACHE II: acute physiology and chronic health evaluation II; Mortality: predicted mortality; SAPS III: simplified acute physiology score III; Sig: significance; SOFA: sequential organ failure assessment; Std: standard *Under the nonparametric assumption ^#^Null hypothesis: true area = 0.5

Test Result Variable(s)	Area	Std. Error*	Asymptotic Sig.^#^	Asymptotic 95% Confidence Interval
				Lower Bound
SOFA	0.741	0.045	0.000	0.653
APACHE II	0.791	0.038	0.000	0.717
APACHE II mortality	0.779	0.038	0.000	0.704
SAPS III	0.831	0.035	0.000	0.763
SAPS III mortality	0.822	0.037	0.000	0.750

## Discussion

Outcome prediction models use several clinical data points to estimate mortality probability and guide patient risk stratification. However, several issues must be considered when evaluating the clinical value of any scoring system.

Although SAPS III demonstrates good discrimination and standard to good calibration, it commonly overestimates mortality [1,2,4,6,11,12]. For example, SAPS III includes variables such as intrahospital location and length of hospital stay before ICU admission, which did not correlate with the primary outcome in this cohort. Another aspect to consider is the fact that SAPS III also takes into account the presence of shock, which, in this cohort, was more frequent in the non-surviving subgroup but was not statistically significant. This inclusion criteria may contribute to higher final scores and the observed overestimation of predicted mortality.

APACHE II demonstrated a good discriminative ability but showed some limitations in calibration, slightly overestimating mortality. Its performance suggests it may have some applicability in IMCUs, particularly in identifying high-risk patients. Previous research supports its adaptation for use in IMCUs, though it requires calibration to improve accuracy [1,2,4,12].

SOFA has been successfully implemented as a mortality prediction score, with performance comparable to other established risk-prediction scores [5,10]. In our cohort, SOFA had the lowest discrimination performance (AUC 0.74). Although organ dysfunction correlates with mortality, it may not be sufficient by itself. Current literature suggests that combining APACHE and SAPS with SOFA results enhances accuracy [5].

While these scores allow quick evaluation and stratification, dichotomizing predictive variables can lead to information loss, increased false negatives, and high dependence on cut-off points [4,5]. These scores were developed and validated in ICU contexts, which differ from IMCU populations. Therefore, extrapolating these scores to IMCU contexts may lead to critical prediction failures.

There are limitations to our analysis. First, our results are based on a single center, and our cohort is relatively small. While a larger-scale, multicenter assessment of each model in the intermediate care setting would be important and necessary for reliable application, we are not aware of large IMCU datasets that reliably include the data elements required for each outcome prediction model [1,2,4,12]. Second, our cohort was created in 2019, and it is uncertain whether the performance we observed would be consistent in a more recent study. Finally, the mortality in our population was lower than the mortality predicted by the models. This aligns with data from previous studies, suggesting that some factors may be affecting the discrimination and calibration of the models assessed in this dataset, which requires further investigation [1,12].

## Conclusions

In a Portuguese medical IMCU, APACHE II and SAPS III scores provided good discrimination for mortality prediction, with SAPS III showing the highest AUC (0,83). However, both scores tend to overestimate mortality, highlighting the need for calibration in the IMCU context. Despite its usefulness, SOFA seems to be a less effective score in IMCUs due to its big emphasis on organ failure. Further research is needed to adapt these models or develop new ones tailored specifically for IMCUs, thereby improving accuracy and clinical usefulness.
